# Therapeutic approach with commercial supplements for pantothenate kinase-associated neurodegeneration with residual PANK2 expression levels

**DOI:** 10.1186/s13023-022-02465-9

**Published:** 2022-08-09

**Authors:** Mónica Álvarez-Córdoba, Diana Reche-López, Paula Cilleros-Holgado, Marta Talaverón-Rey, Irene Villalón-García, Suleva Povea-Cabello, Juan M. Suárez-Rivero, Alejandra Suárez-Carrillo, Manuel Munuera-Cabeza, Rocío Piñero-Pérez, José A. Sánchez-Alcázar

**Affiliations:** Centro Andaluz de Biología del Desarrollo (CABD-CSIC-Universidad Pablo de Olavide), and Centro de Investigación Biomédica en Red: Enfermedades Raras, Instituto de Salud Carlos III, 41013 Sevilla, Spain

**Keywords:** Pantothenate kinase, Pantothenate kinase-associated neurodegeneration, Coenzyme A, Acyl carrier protein, Pantothenate, Pantethine, Vitamin E, Omega 3, Carnitine, Thiamine

## Abstract

**Background:**

Neurodegeneration with brain iron accumulation (NBIA) is a group of rare neurogenetic disorders frequently associated with iron accumulation in the basal nuclei of the brain characterized by progressive spasticity, dystonia, muscle rigidity, neuropsychiatric symptoms, and retinal degeneration or optic nerve atrophy. Pantothenate kinase-associated neurodegeneration (PKAN) is one of the most widespread NBIA subtypes. It is caused by mutations in the gene of pantothenate kinase 2 (PANK2) that result in dysfunction in PANK2 enzyme activity, with consequent deficiency of coenzyme A (CoA) biosynthesis, as well as low levels of essential metabolic intermediates such as 4′-phosphopantetheine, a necessary cofactor for essential cytosolic and mitochondrial proteins.

**Methods:**

In this manuscript, we examined the therapeutic effectiveness of pantothenate, panthetine, antioxidants (vitamin E and omega 3) and mitochondrial function boosting supplements (L-carnitine and thiamine) in mutant PANK2 cells with residual expression levels.

**Results:**

Commercial supplements, pantothenate, pantethine, vitamin E, omega 3, carnitine and thiamine were able to eliminate iron accumulation, increase PANK2, mtACP, and NFS1 expression levels and improve pathological alterations in mutant cells with residual PANK2 expression levels.

**Conclusion:**

Our results suggest that several commercial compounds are indeed able to significantly correct the mutant phenotype in cellular models of PKAN. These compounds alone or in combinations are of common use in clinical practice and may be useful for the treatment of PKAN patients with residual enzyme expression levels.

**Supplementary Information:**

The online version contains supplementary material available at 10.1186/s13023-022-02465-9.

## Background

The term Neurodegeneration with Brain Iron Accumulation (NBIA) refers to a group of rare hereditary neurodegenerative diseases frequently associated with iron accumulation in basal ganglia [1, 2]. The clinical symptoms include dystonia, spasticity, bradykinesia, postural instability, loss of ambulation, loss of speech, dysphagia, psychiatric symptoms, intellectual disability and visual impairment. Currently, 15 genes have been identified to cause the main clinical entities of NBIA [3]. However, the causative mutation is unknown in 20% of cases [4]. Approximately, 50% of cases of NBIA are caused by mutations in the gene of pantothenate kinase 2 (PANK2) which encodes an essential enzyme in coenzyme A (CoA) biosynthesis [5]. The clinical entity caused by PANK2 mutations is termed pantothenate kinase-associated neurodegeneration (PKAN). Although there are several isoforms, PANK1a, PANK1b, PANK2 and PANK 3, only the PANK2 deficiency is associated with PKAN. PANK2 enzyme is localized in mitochondrial intermembrane space and it catalyzes the transformation of (R)-pantothenate into (R)-4’-phosphopantothenate using ATP.

The enzyme alteration causes coenzyme A deficiency, mitochondrial dysfunction and low energy production, intracellular iron accumulation and impaired protection against oxidative damage, which provokes lipid peroxidation in cell membranes, and eventually cell death [4, 6]. Altered mitochondrial membrane potential and deficient mitochondrial respiration have been demonstrated in PANK2-defective neurons derived from KO mice [7] and in cellular models derived from PKAN patients [8–10]. However, the exact pathological mechanisms involved in PKAN are still unclear.

Apart from metabolic alterations including impairment of the Krebs cycle, steroidogenesis, heme biosynthesis, amino acid synthesis, and β-oxidation [11], low CoA levels particularly in mitochondria also affect the 4′-phosphopantetheinylation of essential proteins for mitochondrial function and cell homeostasis [12, 13]. The explanation is that CoA provides the 4'-phosphopantetheine moiety needed for the posttranslational 4'-phosphopantetheinylation modification required to activate specific proteins. Thus, multi-enzyme complexes which sequentially catalyse several reactions are often dependent on the covalent binding of a 4’-phosphopantetheine cofactor to specific proteins. This protein carries metabolic intermediates in the process of different enzymatic reactions. In mammals, the transfer of the 4’-phosphopantetheinyl cofactor from coenzyme A to specific proteins takes a post-translational modification place following protein biosynthesis [14]. Thus, 4’-phosphopantetheinylation is necessary for the conversion of apoenzymes into their full-active forms [14].

Previously, we showed that impaired CoA homeostasis due to PANK2 mutations leads to decreased expression levels of the PANK2 enzyme itself as well as essential mitochondrial 4’-phosphopantetheinyl-proteins such as mtACP which participates in lipoic acid biosynthesis, and consequently affects protein lipoylation and activity of lipoylated proteins including pyruvate dehydrogenase (PDH) [12]. Furthermore, mtACP deficiency was associated with reduced mitochondrial complex I activity and down-regulation of proteins forming the Fe/S cluster synthesis complex [12]. These findings support the hypothesis that PANK2 mutations dramatically alter mitochondrial function affecting the expression levels of mitochondrial phosphopantetheinyl-proteins. Therefore, expression levels of PANK2 and mtACP can be excellent biomarkers to address disease severity and effectiveness of potential treatments. Our results and those obtained by other authors suggest that alterations in mitochondrial metabolism such as lipoic acid synthesis, complex I assembly and Fe-S cluster biogenesis may underlie the molecular pathomechanisms of PKAN [12, 13]. Interestingly, pantothenate can rescue PANK2 and all pathological alterations including mtACP expression levels, PDH and complex I activity, and the expression of Fe-S cluster proteins in responder mutations with residual expression levels of the enzyme [12]. However, pantothenate has no effect in cells harboring stop codon mutations encoding a truncated PANK2 protein which is quickly degraded by the quality control of the cell.

The goal of this work was to address the potential effectiveness of pantothenate and pantothenate-derivatives (panthetine), antioxidants (omega 3 and vitamin E) and mitochondrial function boosting agents (carnitine and thiamine) in correcting the pathological alterations in cellular models of PKAN with residual PANK2 expression levels. The compounds were evaluated individually. The identification of commercial and safe supplements capable of increasing the expression of the mutant enzyme and correcting the molecular alterations downstream of the enzyme defect can be important for making optimal therapeutic decisions in PKAN.

## Material and methods

### Reagents

Monoclonal anti-actin antibody, Prussian Blue, pantothenate, pantethine, vitamin E, L-carnitine, thiamine, Luperox^®^ and trypsin were purchased from Sigma Chemical Co. (St. Louis, MO). Anti-mitochondrial acyl carrier protein (mtACP), anti-NF-Y, anti-FOXN4 and anti-hnRNPA/B were purchased from Invitrogen/Molecular Probes (Eugene, OR). Anti-phospho-PGC1α was purchased from RD systems. NFS1 antibody and Omega 3 were purchased from Santa Cruz Biotechnology (Santa Cruz, CA). Anti-PANK2, anti-PGC1α, complex 1 activity kit and PDH activity kit were purchased from Abcam (Cambridge, UK). Anti-TFAM was purchased from Cell Signaling. BODIPY^®^ 581/591 C11 was purchased from Thermo-Fisher (Waltham, MA). A cocktail of protease inhibitors (complete cocktail) was purchased from Boehringer Mannheim (Indianapolis, IN). The Immun Star HRP substrate kit was from Bio-Rad Laboratories Inc. (Hercules, CA).

### Cells

We used primary skin fibroblasts from two unaffected subjects (control 1, 2) and five patients from the Movement Disorder Unit of Hospital Universitario Virgen del Rocío, Sevilla, Spain. One patient (P1,) is compound heterozygous carrier of changes c.[747dup] that causes a frameshift (p.Arg249Profs) mutation triggering a premature stop codon and c.[1475C > T] (p.Ala492Gly) that causes a missense mutation which is predicted to be damaging by prediction tools such as PolyPhen2 [15]. The second patient (P2) is compound heterozygous carrier of changes in position c. [240_241del] and c.[650C > T] (p.Asp217Gly) which have been previously described [16]. The third patient (P3) carries a compound heterozygous in position c. [950G > C], [1231G > A] (p.[Gly317Ala];[Gly411Arg]). The fourth patient is homozygous carrier of changes in position c.680A > G (p.Y227C), a prevalent mutation in Dominican Republic (with prevalence 1000 times higher than normal) [17]. The fifth patient (P5) carries a homozygous mutation c.1259delG causing a frameshift p.F419fsX472 that result in a truncated PANK2 protein [18].

Control values represent means ± SD for two control fibroblast cell lines. Fibroblasts were grown in DMEM (Sigma) supplemented with 10% FBS (Sigma), 100 mg/ml streptomycin, 100 U/ml penicillin and 4 mM l-glutamine (Sigma). All the experiments were performed with fibroblasts cell cultures with a passage number < 10.

### Ethical statements

Approval of the ethical committee of the Hospital Universitario Virgen Macarena y Virgen del Rocío de Sevilla (Spain) was obtained, according to the principles of the Declaration of Helsinki and all the International Conferences on Harmonization and Good Clinical Practice Guidelines.

### Screening protocol

Our group has developed a personalized drug screening protocol in dermal fibroblasts derived from patients with PKAN. This cell type accumulates iron as it happens at the neuronal level. In this way, variations in intracellular iron levels as a consequence of multiple treatments, doses and times, make it possible to select the drugs capable of eliminating intracellular iron in a personalized way in each patient. For drug screening in PKAN fibroblasts, iron accumulation was determined using a modified Prussian Blue Stain protocol [8]. Fibroblasts from controls or patients were seeded in 6-well or 12-well culture plates. Later, when fibroblasts reach a confluence of 75%, they were treated with different concentrations of the compounds to be tested for 20 days. In a third phase, the analysis of the culture cells was carried out to determine the accumulated intracellular iron in the untreated and treated cultures and the effectiveness of the tested compound for the reduction or reversal of the intracellular iron accumulation was determined by Prussian Blue staining and brightfield microscopy [19]. Perls' staining was quantified in a microplate reader (Polar star Omega, BMG Labtech) and by light microscopy. Images and quantification analysis from light and fluorescence microscopy were performed by using the ImageJ software.

### Iron determination by inductively coupled plasma mass spectrometry (ICP-MS) assays

Iron levels in cell extracts were also determined by ICP-MS [20]. Calibration was performed for six standards and the correlation coefficients (r) ranged from 0.98 to 0.99. Elemental concentrations are shown in µg  Fe^2+^/μg protein. Values are shown as means ± SD (standard deviation) for three independent experiments.

### Immunoblotting

Western blotting was performed using standard Methods described in previous manuscripts of the research group [8]. After protein transfer, membranes were incubated with various primary antibodies diluted 1:1000, and then with the corresponding secondary antibody coupled to horseradish peroxidase at a 1:10,000 dilution. Specific protein complexes were identified using the Immun Star HRP substrate kit (Biorad Laboratories Inc., Hercules, CA, USA). Protein loading was assessed by Ponceau staining and actin expression levels. If the molecular weight of proteins did not interfere, membranes were re-probed with different antibodies. In the case of proteins with different molecular weights, membranes were cut and incubated with specific antibodies.

### Complex I activity

Complex I activity in whole cells was measured using the Complex I Enzyme Activity Dipstick Assay Kit (ab109720, ABCAM, Cambridge, MA, USA) according to manufacturer’s instructions. Three biological replicates were used per measurement. Results are expressed as enzyme activity respect to control. The signal intensity was analyzed by a Molecular Imager ChemiDoc XRS + System (Bio-Rad Laboratories Inc., USA).

### PDH activity

PDH complex activity in whole cells was measured using the Pyruvate dehydrogenase (PDH) Enzyme Activity Dipstick Assay Kit (ab109882, ABCAM, Cambridge, MA, USA) according to manufacturer’s instructions. Three biological replicates were used per measurement. Results are expressed as enzyme activity respect to control. The signal intensity was analyzed by a Molecular Imager ChemiDoc XRS + System (Bio-Rad Laboratories Inc., USA).

### Measurement of membrane lipid peroxidation

Lipid peroxidation was evaluated using 4,4-difluoro-5-(4-phenyl-1,3-butadienyl)-4-bora-3a,4a-diaza-s-indacene-3-undecanoic acid (BODIPY^®^ 581/591 C11), a lipophilic fluorescent dye [21, 22]. Cells were incubated with 1–5 µM BODIPY^®^ 581/591 C11 for 30 min at 37 °C. 500 µM Luperox^®^ for 15 min were used as positive control of lipid peroxidation. Lipid peroxidation in fibroblasts was evaluated by an Axio Vert A1 fluorescence microscope with a 20X objective. Images were analysed with Fiji-ImageJ software.

### Real-time quantitative PCR

Expression of PANK2 gene in fibroblasts was analysed by real time quantitative PCR using mRNA extracts. mRNA was extracted by using standard methods and SYBR Green protocol as a method designed to detect accurate quantification of gene expression and RT-PCR reactions. PANK2 primers used 5’-TTCCCACTCATGACATGCCT-3’ (Forward primer) and 5’-GTGACCGTCCATTGAATCCG-3’ (Reverse primer) amplifying a sequence of 215 nucleotides. Actin was used as a housekeeping control gene and the primers were 5’- AGAGCTACGAGCTGCCTGAC -3’ (Forward primer) and 3’- AGCACTGTGTTGGCGTACAG -5’ (reverse primer).

### Statistical analysis

We used non-parametric statistics that do not have any distributional assumption, given the low reliability of normality testing for small sample sizes used in this work. To compare parameters between groups, variables were evaluated using Mann–Whitney test for two groups and Kruskal–Wallis test to compare multiple groups. All results are expressed as mean ± SD of 3 independent experiments and a p-value < 0.05 was considered as statistically significant. Statistical analyses were made with GraphPad Prism 7.0 (GraphPad Software, San Diego, CA USA).

## Results

### Pantothenate, pantethine, vitamin E, omega 3, L-carnitine or thiamine treatment partially reduce iron accumulation and increase PANK2 and mtACP expression levels in mutant PKAN cells with residual PANK2 expression

**Fig. 1:**
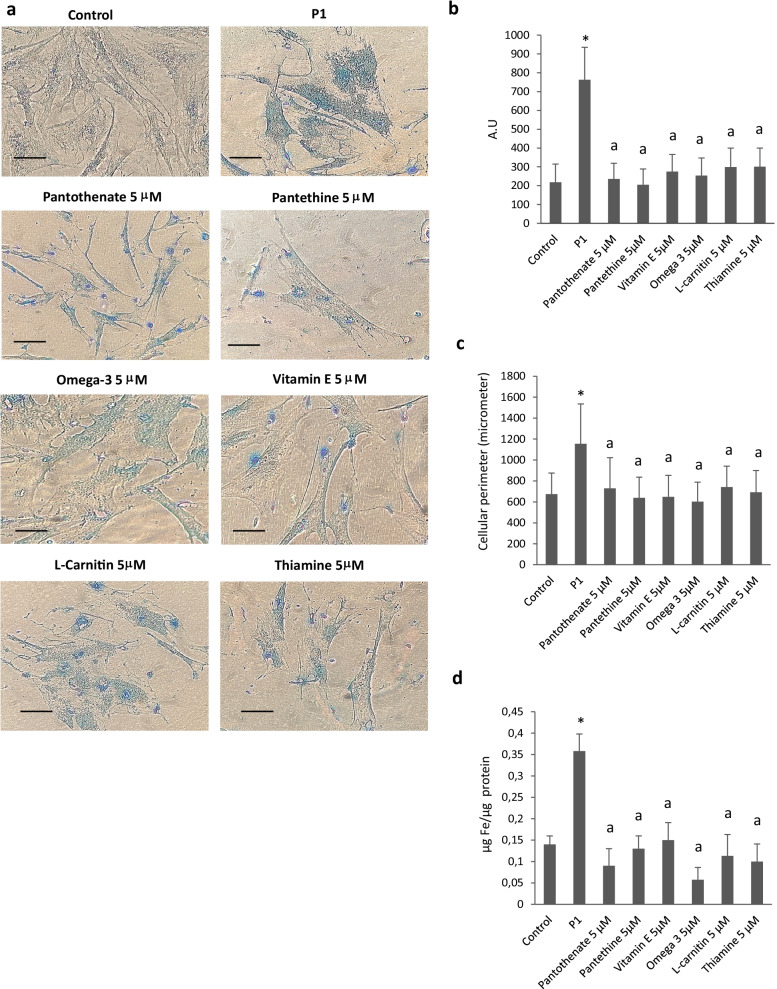
Effect of pantothenate, pantethine, vitamin E, omega 3, L-carnitine or thiamine treatment on iron accumulation and cell morphology in mutant PANK2 cells with residual PANK2 expression levels. **a** Control (C1) and PKAN fibroblasts (P1) were treated with pantothenate, pantethine, vitamin E, omega 3, L-carnitine or thiamine at 5 μM for 20 days. Then, cells were stained with Prussian Blue as described in Material and Methods and examined by bright-field microscopy. Scale bar = 15μm. **b** Quantification of Prussian Blue staining Images were analyzed by the Image J software. **c** Cell perimeter of untreated and treated Control and PKAN fibroblasts (P1). Images were analyzed by the Image J software. **d** Total iron content of untreated and treated control and PKAN cells was determined by ICP-MS as described in Material and Methods. Data represent the mean ± SD of three separate experiments. *p < 0.01 between Control and PKAN fibroblasts. ^a^*p* < 0.01 between untreated and treated fibroblasts. A.U., arbitrary units

First, we identified six compounds able to eliminate intracellular iron accumulation and senescent cell morphology in patient P1 harboring a double heterozygous mutation (one allele harbors a stop codon mutation and the other a missense mutation) with residual levels of PANK2 protein. As shown in Fig. 1a, b and c pantothenate, pantethine, vitamin E, omega 3, L-carnitine and thiamine at 5 µM significantly reduced Prussian Blue staining and normalized cell morphology in P1 fibroblasts. Iron accumulation and elimination by positive compounds in mutant PANK2 cells were corroborate by ICP-MS assays (Fig. 1d).

**Fig. 2:**
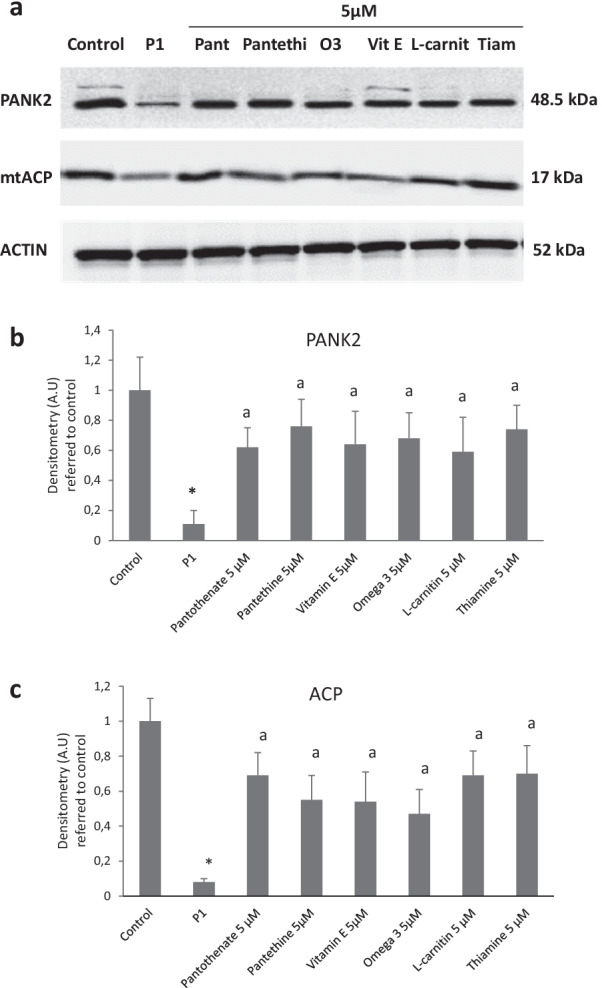
Effect of pantothenate, pantethine, vitamin E, omega 3, L-carnitine or thiamine treatment on PANK 2 and mtACP expression levels in mutant PANK2 cells with residual PANK2 expression levels. **a** Control (C1) and PKAN fibroblasts (P1) were treated with pantothenate (Pant), pantethine (Pantethi), vitamin E (Vit E), omega 3 (O3), L-carnitine (L-carnit) or thiamine (Tiam) at 5 μM for 20 days. Protein extracts (50 μg) were separated on a SDS polyacrylamide gel and immunostained with antibodies against PANK2 and mtACP. Actin was used as a loading control. **b** Densitometry of the Western blotting of PANK2. **c** Densitometry of the Western blotting of mtACP. Data represent the mean ± SD of three separate experiments. *p < 0.01 between PKAN patients and controls. ^a^*p* < 0.01 between untreated and treated fibroblasts. A.U., arbitrary units

Next, to examine if any residual PANK2 enzyme could be stabilized by the beneficial compounds in PKAN fibroblasts, control and affected cells were treated with pantothenate, pantethine, vitamin E, omega 3, L-carnitine or thiamine and expression levels of PANK2 and mtACP were evaluated. As is illustrated in Fig. 2a, b and c, all positive compounds correcting iron accumulation and cell morphology were also able to increase PANK2 and mtACP expression levels (Fig. 2a, b and c).

### Dose response effect of positive compounds (pantothenate, pantethine, vitamin E, omega 3, L-carnitine or thiamine) on PANK2 and mtACP expression levels

We then examined the effect of a dose–response assay (1–100 μM) of the six positive compounds (pantothenate, pantethine, vitamin E, omega 3, L-carnitine or thiamine) on PANK2 and mtACP expression levels by Western blotting. In addition, as mtACP also participates in Fe/S cluster biosynthesis (20), we also explored the expression levels of NFS1 which participates in the mitochondrial Fe/S cluster synthesis complex.

**Fig. 3:**
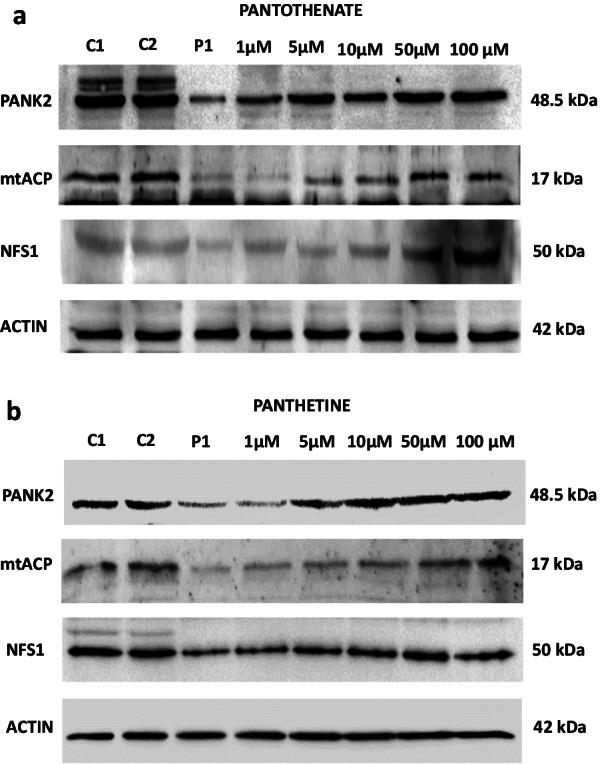
Dose response effect of pantothenate or pantethine treatment on PANK2, mtACP and NFS1 expression levels in mutant PANK2 cells with residual PANK2 expression levels. PKAN fibroblasts (P1) were treated with increasing concentrations of pantothenate **a** or pantethine **b** at 1, 5, 10, 50 and 100 μM for 20 days. Protein extracts (50 μg) were separated on a SDS polyacrylamide gel and immunostained with antibodies against PANK2, mtACP and NFS1. Actin was used as a loading control

**Fig. 4:**
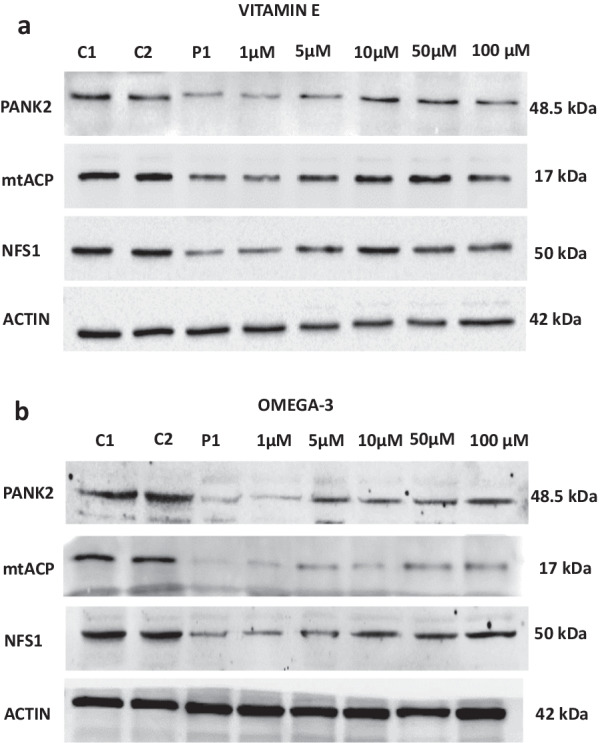
Dose response effect of vitamin E or omega 3 treatment on PANK2, mtACP and NFS1 expression levels in mutant PANK2 cells with residual PANK2 expression levels. PKAN fibroblasts (P1) were treated with increasing concentrations of vitamin E **a** or omega 3 **b** at 1, 5, 10, 50 and 100 μM for 20 days. Protein extracts (50 μg) were separated on a SDS polyacrylamide gel and immunostained with antibodies against PANK2, mtACP and NFS1. Actin was used as a loading control

**Fig. 5:**
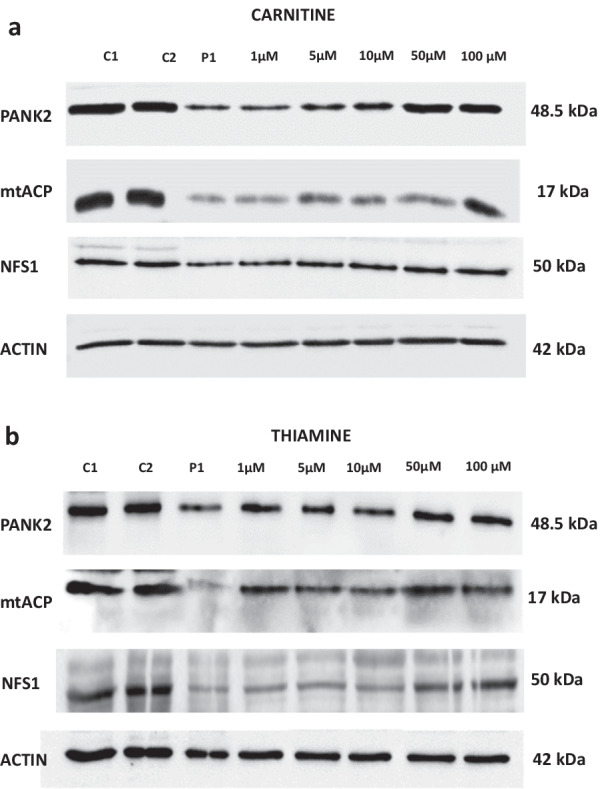
Dose response effect of carnitine or thiamine treatment on PANK2, mtACP and NFS1 expression levels in mutant PANK2 cells with residual PANK2 expression levels. PKAN fibroblasts (P1) were treated with increasing concentrations of carnitine **a** or thiamine (**a**) at 1, 5, 10, 50 and 100 μM for 20 days. Protein extracts (50 μg) were separated on a SDS polyacrylamide gel and immunostained with antibodies against PANK2, mtACP and NFS1. Actin was used as a loading control

The six selected compounds showed a dose response positive effect in PANK2, mtACP and NFS1 expression levels (Figs. 3a, b, 4a, b, 5a and b; Additional file 1: Figs S1, S2 and S3). The positive effect was noticeable since 1–5 μM and reached a maximum effect at 50–100 μM.

The effect of the different treatments (pantothenate, pantethine, vitamin E, omega 3, L-carnitine or thiamine) on control cells is shown in Additional file 1: Fig S4a, b.

### Positive compounds (pantothenate, pantethine, vitamin E, omega 3, L-carnitine or thiamine) up-regulate PANK2 gene transcription and increase the expression levels of essential transcription factors

**Fig. 6:**
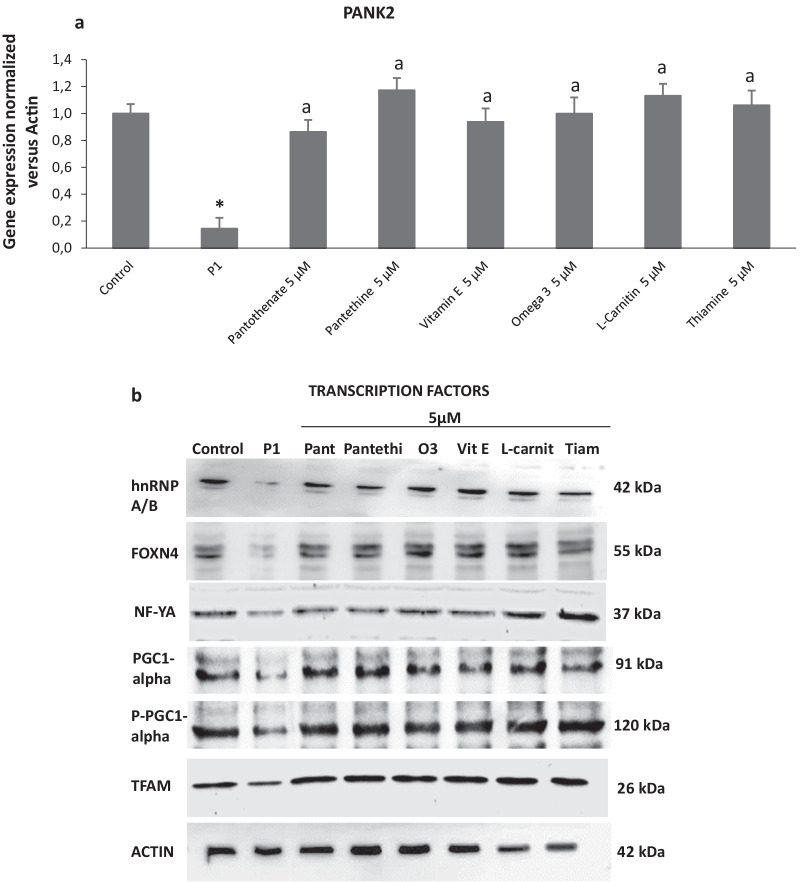
Effect of pantothenate, pantethine, vitamin E, omega 3, L-carnitine or thiamine treatment on PANK2 transcripts. **a** Control (C1) and patient P1 fibroblasts were treated with pantothenate, pantethine, vitamin E, omega 3, L-carnitine or thiamine at 5 μM for 20 days. PANK2 transcripts were quantified by qPCR as described in Material and Methods. **b** Protein expression levels of transcription factors NF-Y, FOXN4, hnRNPA/B, PGC-1alpha, PPGC-1 alpha and TFAM assessed by Western blotting. Data represent the mean ± SD of three separate experiments. *p < 0.01 between PKAN patients and controls. ^a^*p* < 0.01 between untreated and treated fibroblasts. A.U., arbitrary units

Furthermore, the favourable effect of the six positive compounds on PANK2 protein expression levels was associated with an increase in the steady-state levels of PANK2 transcripts (Fig. 6a) suggesting that all six beneficial compounds up-regulated PANK2 gene expression or transcript stabilization. Indeed, the six compounds were able to increase the expression levels of well-known transcription factors that binds the PANK2 promoter such as NF-Y, FOXN4 and hnRNPA/B (Fig. 6b and Additional file 1: Fig S5) [23]. These results support the hypothesis that favourable compounds increased the transcription of the PANK2 gene.

We also examined protein expression levels of PGC-1alpha, PPGC1alpha and TFAM which are members of a family of transcription coactivators that play a central role in the regulation of cellular energy metabolism [24–26]. PGC-1alpha stimulates mitochondrial biogenesis and participates in the regulation of both carbohydrate and lipid metabolism while PPGC-1alpha is the active form of PGC-1alpha. TFAM plays a role in the determination of mitochondrial genome by regulating packaging, stability and replication. Therefore, disruption of TFAM could lead to mtDNA depletion and deficient mitochondrial bioenergetics. The six positive compounds were able to restore the decreased expression levels of PGC-1alpha, PPGC-1alpha and TFAM in P1 cells with residual PANK2 expression levels (Fig. 6b and Additional file 1: Fig S5).

### Positive compounds (pantothenate, pantethine, vitamin E, omega 3, L-carnitine or thiamine) reduce lipid peroxidation of affected cells

**Fig. 7:**
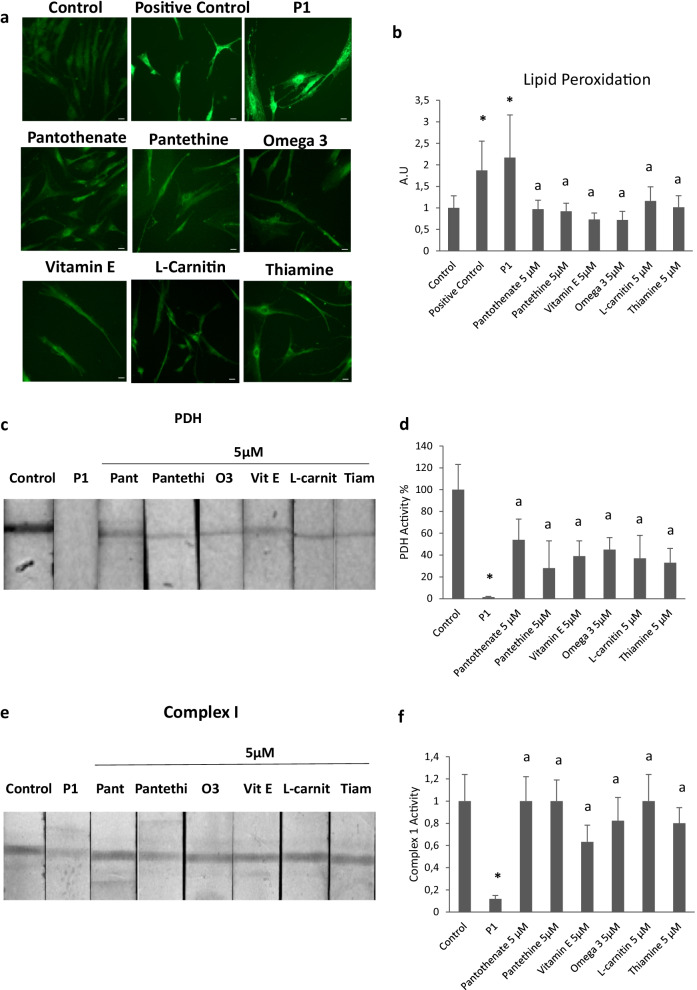
Effect of pantothenate, pantethine, vitamin E, omega 3, L-carnitine or thiamine treatment on lipid peroxidation, PDH activity and mitochondrial complex I activity in mutant PANK2 cells with residual PANK2 expression levels. P1 fibroblasts were treated with pantothenate, pantethine, vitamin E, omega 3, L-carnitine or thiamine at 5 μM for 20 days. **a** Lipid peroxidation was assessed by BODIPY staining and fluorescence microscopy analysis. **b** Quantification of BODIPY signal. **c** PDH activity in whole cellular extracts was determined as described in Material and Methods. **d** Quantification of PDH activity. **e** Mitochondrial complex I activity in whole cellular extracts was determined as described in Material and Methods. **f** Quantification of Complex I activity Data represent the mean ± SD of three separate experiments. *p < 0.01 between PKAN patients and controls; ^a^*p* < 0.01 between untreated and treated cells

Literature supports the evidence of the relation between iron, reactive oxygen species (ROS) production and lipid peroxidation [27–30]. As consequence of increase intracellular iron in PKAN cells, the Fenton reaction may occur and generates high levels of ROS, which damage lipids through peroxidation [27]. With the aim to confirm whether lipid peroxidation is a secondary pathological event in PKAN cells and to assess the effect of favorable compounds, treated and untreated mutant cells were stained with Bodipy, a fluorescent radio-probe for indexing lipid peroxidation and antioxidant efficacy in model membrane systems and living cells. As is shown in Fig. 7a and b, PKAN cells showed increased levels of lipid peroxidation respect to control cells. Interestingly, all six positive compounds reduced lipid peroxidation in mutant cells.

### Positive compounds (pantothenate, pantethine, vitamin E, omega 3, L-carnitine or thiamine) also correct PDH and complex I activity in PKAN cells with residual PANK2 levels

Next, we focused in the pathological alterations potentially induced by mtACP deficiency. Thus, mtACP is necessary for lipoic acid synthesis by mitochondrial FAS II [31]. Furthermore, lipoic acid is a crucial sulfur-containing cofactor in cellular metabolism [32, 33]. As a lysine posttranslational modification on particular components of enzymatic complexes, this functional group is required for the activities of these multimeric complexes [34, 35]. For example, the pyruvate dehydrogenase (PDH) and alpha-ketoglutarate (KDH) complexes regulate carbon entry points into the central metabolic pathway of the tricarboxylic acid cycle (TCA) [36]. On both complexes, lipoylation is critical for proper enzyme function, and deficiency of this modification inhibits their activities.

As shown in Fig. 7c and d, PDH activity was markedly reduced in PKAN fibroblasts. Interestingly, all six supplements (pantothenate, pantethine, vitamin E, omega 3, L-carnitine or thiamine at 5 μM) were able to restore partially PDH activity (Fig. 7c and d) in responder mutant PANK2 fibroblasts.

As mtACP is also critically involved in the assembly of mitochondrial respiratory complex I [37], we next evaluated complex I activity in control and PANK2 mutant fibroblasts. The activity of complex I was significantly reduced in mutant fibroblasts (Fig. 7e and f). The restoration of PANK2 and mtACP expression levels by the six positive compounds was also able to restore complex I enzymatic activity (Fig. 7e and f).

### Positive compounds (pantothenate, pantethine, vitamin E, omega 3, L-carnitine or thiamine) increased PANK2 and mtACP expression levels in several PKAN cell lines with residual PANK2 levels

**Fig. 8:**
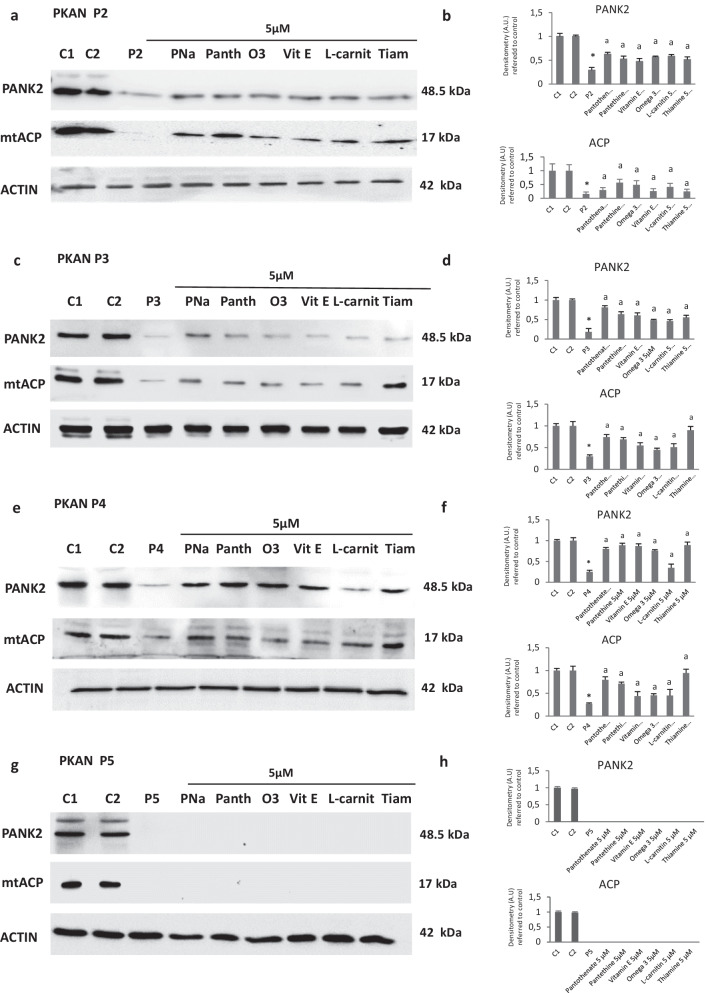
Effect of pantothenate, pantethine, vitamin E, omega 3, L-carnitine or thiamine treatment on PANK 2 and mtACP expression levels in mutant PANK2 cells with residual PANK2 expression levels and mutant cells with expression of truncated PANK2. PKAN fibroblasts were treated with pantothenate, pantethine, vitamin E, omega 3, L-carnitine or thiamine at 5 μM for 20 days. Protein extracts (50 μg) were separated on a SDS polyacrylamide gel and immunostained with antibodies against PANK2 and mtACP. Actin was used as a loading control. **a** Western blotting of mutant of P2 fibroblasts. **b** Densitometry of Western blotting of mutant P2 fibroblasts. **c** Western blotting of mutant of P3 fibroblasts. **d** Densitometry of Western blotting of mutant P3 fibroblasts. **e** Western blotting of mutant of P4 fibroblasts. **f** Densitometry of Western blotting of mutant P4 fibroblasts. **g** Western blotting of mutant of P5 fibroblasts. **h** Densitometry of Western blotting of mutant P5 fibroblasts. Data represent the mean ± SD of three separate experiments. *p < 0.01 between PKAN patients and controls. ^a^*p* < 0.01 between untreated and treated fibroblasts. A.U., arbitrary units

Next, we examined the effectiveness of beneficial compounds at 5 μM in three additional cell lines (P2, P3 and P4) carrying mutations with residual protein expression levels (Fig. 8a–f) and in one cell line (P5) harboring a homozygous mutation causing a truncated PANK2 protein. As expected, positive compounds were able to increase the expression levels of PANK2 and mtACP in mutant cells lines with residual expression of PANK2 (Fig. 8a–f) but had no effect in mutant cells with a truncated PANK2 protein (Fig. 8g, h). Furthermore, the increased levels of PANK2 and mtACP induced by positive compounds were associated with reduced iron accumulation in mutant cells with residual PANK2 levels but not in mutant cells with truncated PANK2 (Additional file 1: Figs S6, S7, S8, S9).

## Discussion

In this manuscript, we show that 6 commercial supplements (pantothenate, pantethine, vitamin E, omega 3, carnitine and thiamine) can eliminate iron accumulation, increase PANK2 and mtACP protein levels and improve pathological alterations in mutant cells with residual PANK2 expression levels.

Although clinical studies are being carried out with several compounds [38], currently existing treatments for PKAN are primarily palliative to pharmacologically treat spasticity, seizures, dystonia, or psychiatric disorders; therefore, this innovative approach to personalized pharmacological screenings may enable more effective drug discovery targeting PANK2 deficiency.

In a previous work, using cellular models derived from PKAN patients, we confirmed the hypothesis that CoA deficiency caused by PANK2 mutations affects the expression levels and activity of key mitochondrial proteins harboring a 4′-phosphopantetheinyl cofactor such as mtACP, ALDH1L2 or AASS [12]. Reduced mtACP levels also affects the lipoylation of pyruvate dehydrogenase (PDH) and α-ketoglutarate dehydrogenase (α-KGDH). Our observations together with the findings of other authors [13] have implications for potential therapeutic approaches for PKAN. In pantothenate-responder mutations a reasonable therapeutic option would be pantothenate treatment that is able to increase PANK expression, correct CoA levels in mitochondria, and normalize the expression levels of mtACP and other phosphopantetheinyl proteins in cellular models of PKAN [8]. This strategy is based on the hypothesis that a functionally weak enzyme may work better with higher concentrations of its substrate. The ability of high-dose pantothenate to overcome a partially impaired PANK enzyme is supported by in vitro studies where the enzyme affinity for pantothenate is lower but the reaction is functional [39]. These in vitro studies are valuable because they demonstrate that high-dose pantothenate may be useful for patients who have some residual PANK2 function. However, this approach is not effective in patients harboring stop codons mutations in both alleles without residual enzyme. For this reason, the evaluation of the effect of pantothenate supplementation in patient-derived cells can provide a useful information about the characteristics of a particular mutation. In addition, it is necessary to assess whether pantothenate supplementation could reach the necessary concentration for functional effects in the human brain in vivo. A strategy to overcome this problem it would be to combine pantothenate with other pantothenate derivatives such as pantethine with the aim of reaching the necessary concentrations in the brain.

Pantethine, a naturally occurring compound synthesized in the body from pantothenic acid via addition of cysteamine, which acts as an intermediate in the production of coenzyme A, can also be a source of pantothenate because it is highly unstable in serum, and it is rapidly converted to pantothenate and cysteamine [40, 41]. Pantethine is a dimeric form of pantetheine that was shown to rescue PKAN disease phenotypes in bacteria [42], Drosophila [43], zebrafish [44] and mouse [45] models. Until now, most studies of pantethine as a potential therapeutic in PKAN have been limited to animal models, although the compound has been utilized as a lipid-lowering agent in previous human studies [46]. A recent open-label trial evaluated the safety and efficacy of 60 mg/day of pantethine in fifteen children with PKAN for twenty-four weeks [47]. Serum CoA levels were not altered before or after treatment, and there was no significant change in the primary endpoints. The limited efficacy of pantethine in affected patients may be due to poor pharmacokinetic properties or a low dose concentration. However, as pantethine can be also a source of pantothenate, the combination of pantothenate and pantethine supplementation may increase more the substrate tissue concentration than the individual treatments.

The oxidative status has been previously analyzed in PKAN fibroblasts [18]. Sign of oxidative stress was detected in cells from patients, and ROS production was increased in these cells after exposure to iron. In agreement with these findings, our group found increased amount of carbonylated proteins and mitochondrial lipid peroxidation in PANK2 mutant fibroblast, which were prevented by the treatment with pantothenate in responder mutant cells [8]. Lipid peroxidation can be described generally as a process under which oxidants such as free radicals or nonradical species attack lipids containing carbon–carbon double bond(s), especially polyunsaturated fatty acids (PUFAs) resulting in lipid peroxyl radicals and hydroperoxides [31]. The process of lipid peroxidation consists of three steps: initiation, propagation, and termination [48]. Extensive information about the chemistry associated with each of these steps is available elsewhere [49]. Multiple breakdown molecules, such as malondialdehyde (MDA) and 4-hydroxynonenal (4-HNE) are produced in this process [50]. Among several substrates, proteins and DNA are particularly susceptible to modification caused by these aldehydes. MDA and 4-HNE adducts play a critical role in multiple cellular processes and can participate in secondary deleterious reactions (e.g., crosslinking) by promoting intramolecular or intermolecular protein/DNA crosslinking that may alter the functional characteristics of biomolecules, which may aggravate the pathophysiology of the disease.

Vitamin E is a well-characterized chain-breaking antioxidant with the particular function of preventing the cyclic propagation of lipid peroxidation in membrane systems [51]. Vitamin E is also essential for neurological function. This fact, together with a growing body of evidence indicating that neurodegenerative processes are associated with oxidative stress, lead to the hypothesis that several neurodegenerative diseases may be prevented and/or relieved by the antioxidant properties of vitamin E [52]. Studies with humans and with animal models of vitamin E deficiency established its critical roles in protecting the brain, and especially the cerebellum, from oxidative damage and motor coordination deficits [53]. The function of vitamin E has been traditionally ascribed to its antioxidant activity. This assumption is based on many reports that demostrated the positive effect of vitamin E in neutralizing unstable lipid peroxy-radicals generated from polyunsaturated fatty acids [54]. Lipid peroxidation is associated with the development of many neurodegenerative disorders, including Alzheimer’s disease (AD), Parkinson’s disease (PD), and amyotrophic lateral sclerosis (ALS), all of which show elevated levels of lipid peroxidation. The PKAN’s pathomechanism is directly related to the overproduction of ROS and unbalanced mitochondrial redox, which may trigger a neuronal death cascade [55]. Particularly, in fibroblast and neuronal cells from derived from of PKAN’s patients, lipid peroxidation and alteration of oxidative status (increased ROS production), mitochondrial impairment (including defects in mitochondrial respiration and electrophysiological properties) and premature cell death have been detected [9, 18]. Thus, the inhibition of lipid peroxidation propagation might slow the progression and reduce the severity of PKAN disease.

The beneficial effects of omega-3 fatty acids supplementation in a great variety of disorders are now well established by many works demonstrating their involvement in multiple biochemical functions, including anti-inflammatory properties, membrane fluidity, intracellular signaling, and gene expression [56–58]. There is accumulating scientific evidence on the possible efficacy of omega-3 fatty acids supplementation in neurodegenerative disorders [59, 60], such as Parkinson’s (PD) and Alzheimer’s disease (AD) [61].

In summary, antioxidants such as vitamin E and omega 3 can be useful molecules to protect cell membranes from lipid peroxidation which is one of the main pathological alterations identified in PKAN [8, 12] and in other NBIA subtypes [62].

On the other hand, carnitine, a well-known dietary supplement which is needed for the translocation of fatty acids into the mitochondrial compartment for β-oxidation and it has a role in carbohydrate metabolism, possesses this potential to raise the mitochondrial biogenesis by increasing various mitochondrial components’ gene expression. Furthermore, carnitine maintains mitochondrial function via supplying their respective substrates and protecting them against insults including toxic products’ or reactive radicals’ accumulation [63, 64]. Thus, defective OXPHOS, as in PKAN, can be associated with impaired β -oxidation, preferentially affecting brain, heart and skeletal muscle. Reduced function of the respiratory chain generates an increased NADH/NAD ^(+)^ ratio that inhibits β-oxidation and produces secondary carnitine deficiency while increasing ROS and depleting alpha-tocopherol [65]. Therefore, L-carnitine as natural compound which can enhance cellular energy transduction may have therapeutical potential in PKAN. Studies in recent years have demonstrated the protective effects of L-carnitine treatment on mitochondrial functions [64].

Furthermore, as PDH deficiency is a pathological characteristic in PKAN, PDH boosting agents such as thiamine [66] may help as complementary therapies. Thiamine has several roles in cellular glucose metabolism as it functions as a cofactor for various enzyme complexes. PDH and α-KGDH enzyme complexes are important thiamine dependent enzyme complexes that help liberate energy from glucose in the citric acid cycle of mitochondria. Thiamine treatment is very effective for some patients with PDH deficiency. Among these patients, five mutations of the pyruvate dehydrogenase (E1) alpha subunit have been reported previously: H44R, R88S, G89S, R263G, and V389fs [67–71].

Interestingly, all the positive supplements identified in our work up-regulate PANK2 transcripts levels and increased key transcription factors, such as NF-Y, FOXN4 and hnRNPA/B, involved in PANK2 gene expression [23]. In addition, favorable supplements activate the expression of the key mitochondrial regulators such as PGC1α and TFAM [24–26]. Altogether, our data provide mechanistic insights into the mechanism of positive effect of pantothenate, pantethine, vitamin E, omega 3, carnitine and thiamine.

Given that the selected supplements are individually positive in PKAN cellular models, an interesting strategy would be to evaluate the individual and combined effect of these compounds in clinical practice. In fact, combination compounds that impact multiple targets simultaneously are better at controlling complex disease systems, are less prone to drug inefficiency and are the practice standard in many important therapeutic areas [72, 73]. The limitations of many monotherapies can be overcome by targeting disease pathomechanisms on multiple fronts [74]. The systematic screening of combination of drugs in vitro can identify these multi-target mechanisms. Personalized screenings in patient-derived cellular model using active pharmaceutical ingredients can be especially valuable because potential synergies identified by these screens can rapidly move into preclinical and clinical development [75]. In addition, combination effects between compounds with known molecular targets can reveal unexpected relationships between disease pathways [76].

Impaired mitochondrial function, excessive oxidative stress in human brain, genetic factors, and malfunction in human brain metabolism contribute to the progression of neurodegenerative diseases [77]. Multitarget therapeutics with antioxidant and mitochondrial boosting compounds hold promise in tackling the multifactorial and complex nature of neurodegenerative diseases such as PKAN [78, 79]. The use of multitarget therapeutics has emerged in the recent years as a powerful strategy in the development of potential therapeutics for neurological disorders.

However, as drug delivery to brain remained a challenge in the treatment of neurodegenerative diseases, further studies about the clinical effects of positive compounds are needed taking into account their bioavailability, pharmacokinetics, and particularly their blood–brain-barrier crossing ability [80].

## Conclusions

In our work we have identified six commercial compounds able to eliminate iron accumulation and increase PANK2 and mtACP expression levels in mutant cells with residual PANK2 activity. The increase expression levels of these proteins were also associated with a significant improvement in the main pathological alterations of PKAN cells.

Pantothenate, pantethine, vitamin E, omega 3, carnitine and/or thiamine supplementation can be of help for the treatment of PKAN patients with PANK2 residual expression levels.

Personalized screenings in cell models derived from patients can be helpful for evaluating the behavior of particular mutations under different therapeutic options and thus select the most effective supplements and dose concentrations considering their pharmacokinetics properties.

## Supplementary Information

**Additional file 1**. Supplementary figures.

## Data Availability

Data and material that support the findings of this study are available under request.

## Abbreviations

AASS: Alpha-aminoadipic semialdehyde synthase
AD: Alzheimer’s disease
ALDH1L2: Mitochondrial 10-formyltetrahydrofolate dehydrogenase
Alpha-KGDH: Alpha-ketoglutarate dehydrogenase
ALS: Amyotrophic lateral sclerosis
ATP: Adenosine triphosphate
CoA: Coenzime A
DMEM: Dulbecco's modified eagle medium
DNA: Deoxyribonucleic acid
FBS: Fetal bovine serum
FOXN4: Forkhead box protein N4
hnRNPA/B: Heterogeneous nuclear ribonucleoprotein A/B
HRP: Horseradish peroxidase
ICP-MS: Inductively coupled plasma mass spectrometry
LPO: Lipid peroxidation
MDA: Malondialdehyde
mtACP: Mitochondrial acyl carrier protein
NADH/NAD +: Nicotinamide adenine dinucleotide
NBIA: Neurodegeneration with brain iron accumulation
NF-Y: Nuclear transcription factor Y
NFS1: Cystine desulfurase
OXPHOS: *Oxidative phosphorylation* system
PANK2: Pantothenate kinase 2
PBS: Phosphate buffer saline
PD: Parkinson’s disease
PDH: Pyruvate dehidrogenase
PGC-1alpha: Peroxisome proliferator-activated receptor-gamma coactivator
PKAN: Pantothenate kinase-associated neurodegeneration
PPGC-1alpha: Phospho Peroxisome proliferator-activated receptor-gamma coactivator
PUFAs: Polyunsaturated fatty acids
ROS: Reactive oxygen species
SDS: Sodium dodecyl sulfate
STF: Streptomycin
TFAM: Transcription factor A
4-HNE: 4-Hydroxynonenal
