# Supplementary material for: Therapeutic approach with commercial supplements for pantothenate kinase-associated neurodegeneration with residual PANK2 expression levels
Source: Orphanet J Rare Dis. 2022 Aug 9;17:311. doi: 10.1186/s13023-022-02465-9 (PMC9364590; doi:10.1186/s13023-022-02465-9)
Supplement: Supplementary file 1 — Additional file 1. Supplementary figures. [file 13023_2022_2465_MOESM1_ESM.docx]

Supplementary Material

## Supplementary Figures


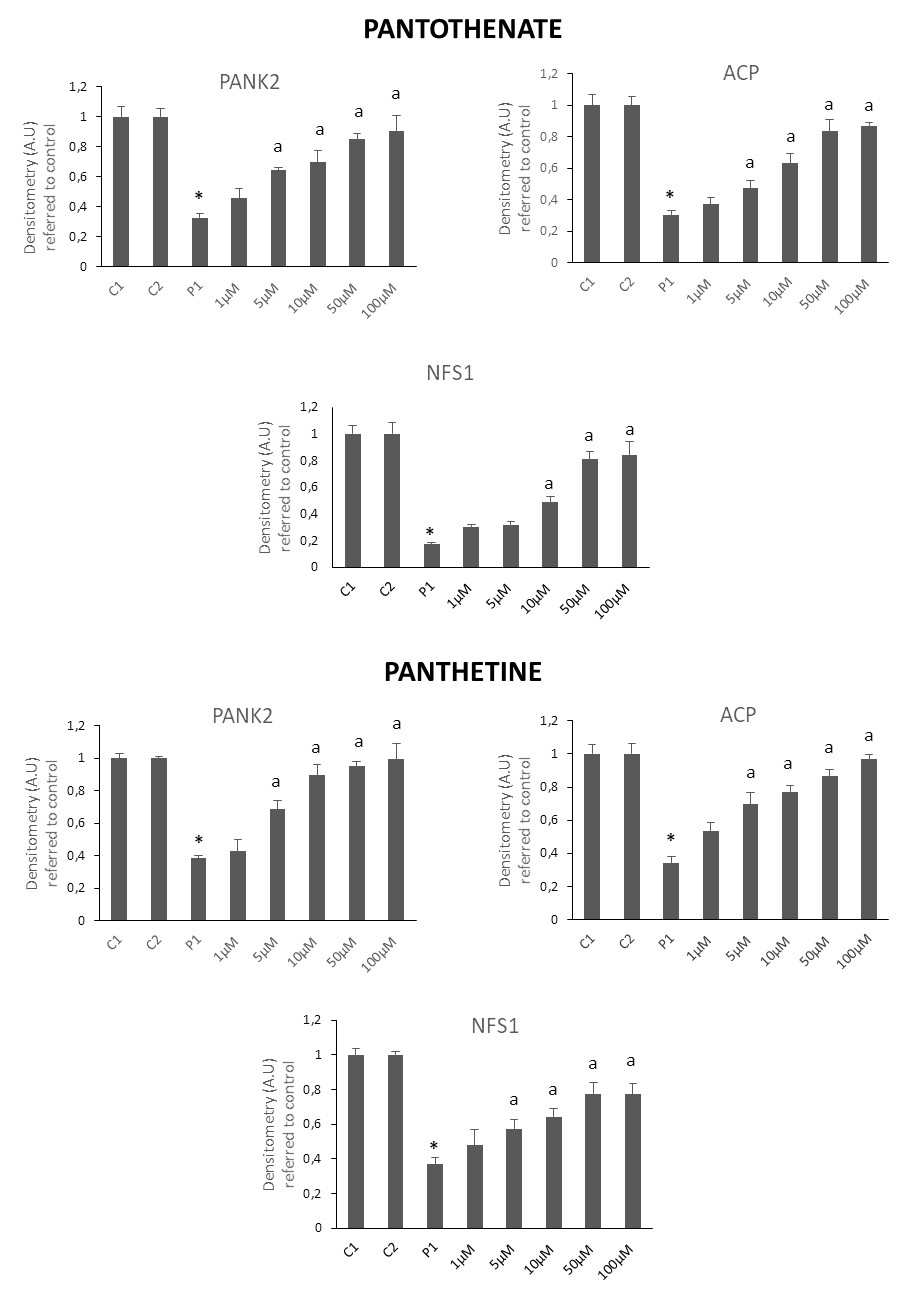


**Supplementary Figure 1.**

Densitometry Westerns Figure **3**. Data represent the mean±SD of three separate experiments. *p<0.01 between PKAN patients and controls. ^a^p<0.01 between untreated and treated fibroblasts. A.U., arbitrary units.

**
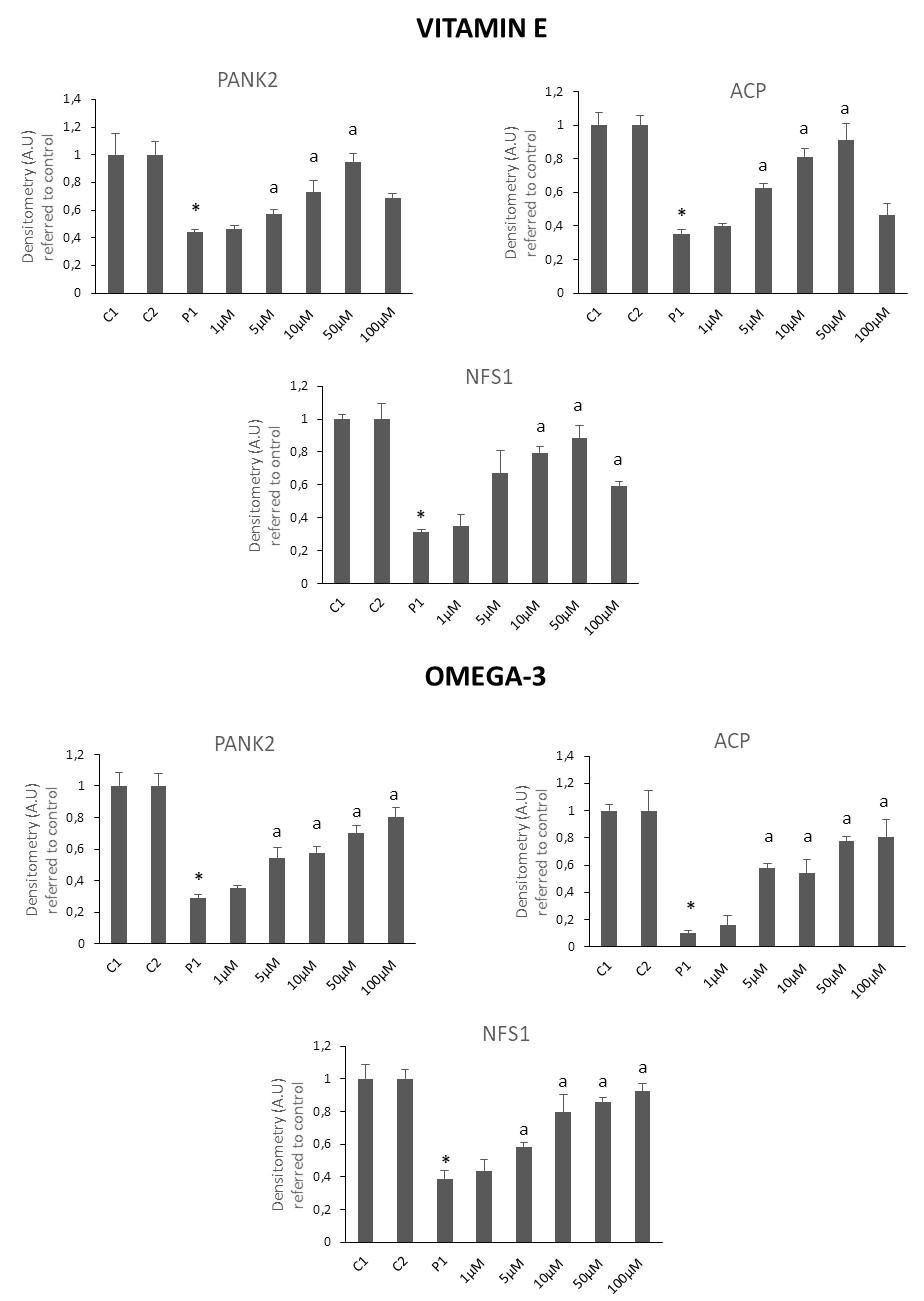
**

**Supplementary Figure 2.**

Densitometry Westerns Figure **4**. Data represent the mean±SD of three separate experiments. *p<0.01 between PKAN patients and controls. ^a^p<0.01 between untreated and treated fibroblasts. A.U., arbitrary units.


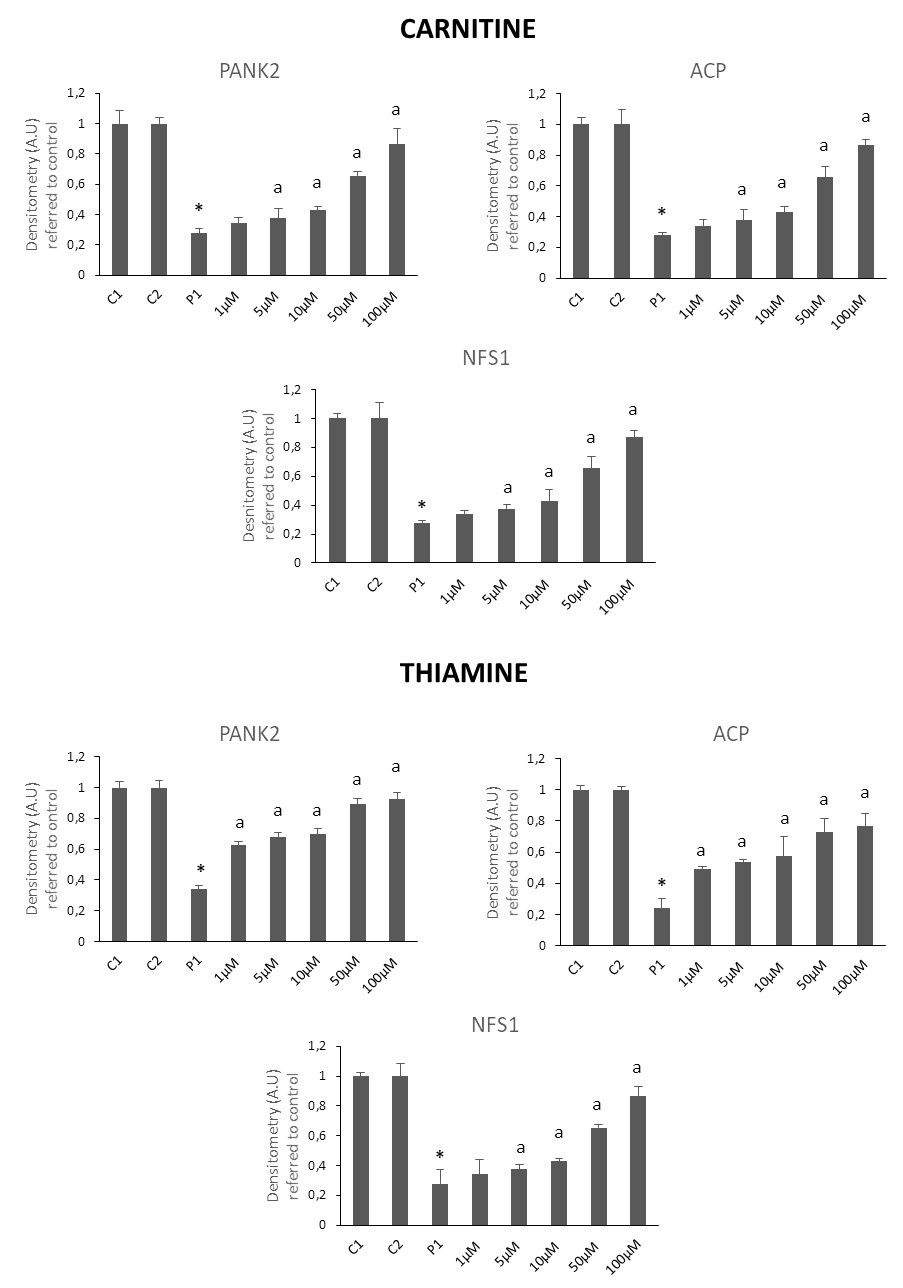


**Supplementary Figure 3.**

Densitometry Westerns Figure **5**. Data represent the mean±SD of three separate experiments. *p<0.01 between PKAN patients and controls. ^a^p<0.01 between untreated and treated fibroblasts. A.U., arbitrary units.

**
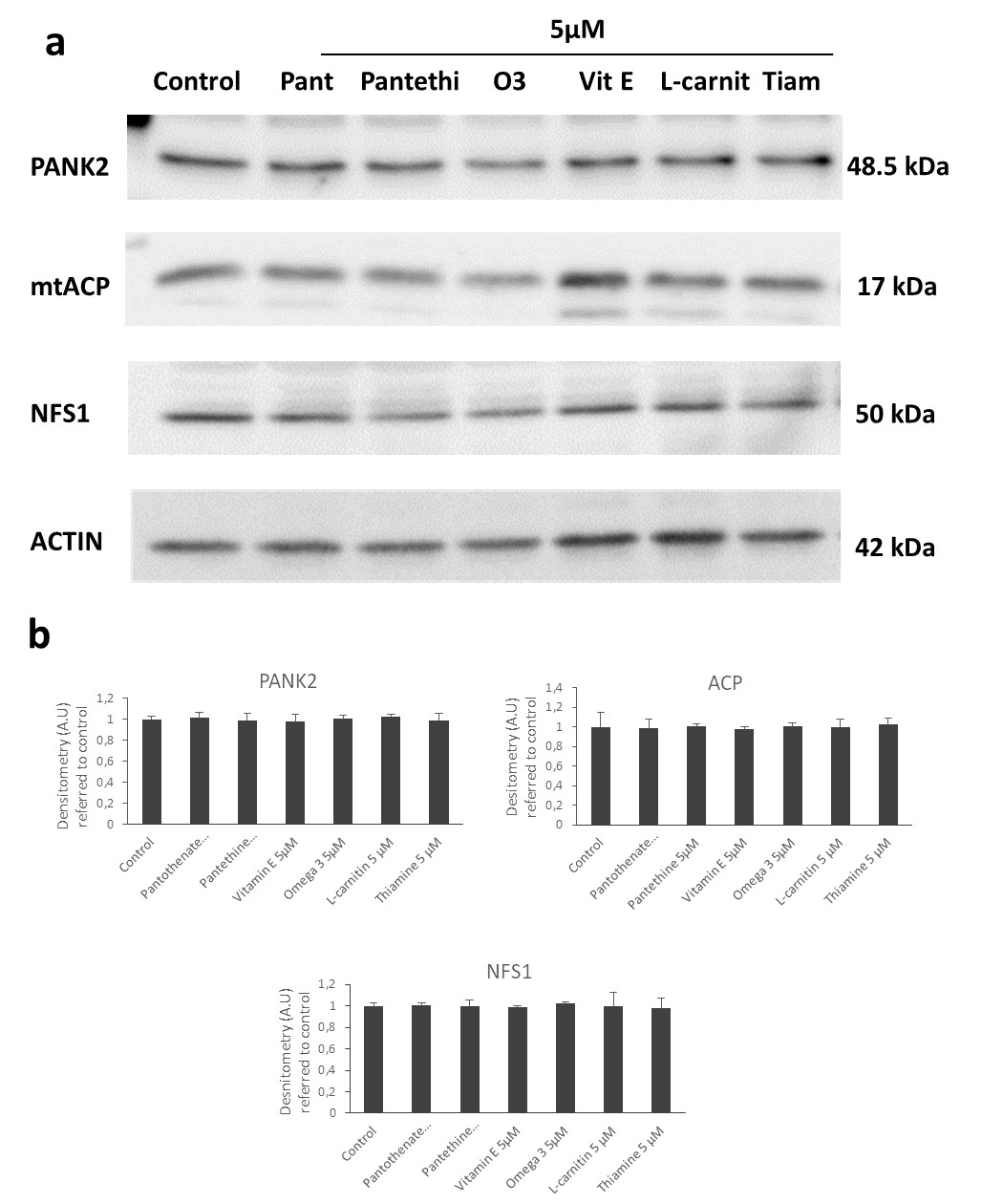
**

**Supplementary Figure 4.**

(**a**) Control cells were treated with pantothenate (Pant), pantethine (Pantethi), vitamin E (Vit E), omega 3 (O3), L-carnitine (L-carnit) or thiamine (Tiam) at 5 μM for 20 days. Protein extracts (50 μg) were separated on a SDS polyacrylamide gel and immunostained with antibodies against PANK2, mtACP and NFS1. Actin was used as a loading control. (**b**). Densitometry of the Western blotting of PANK2. Data represent the mean±SD of three separate experiments. *p<0.01 between PKAN patients and controls. ^a^p<0.01 between untreated and treated fibroblasts. A.U., arbitrary units.


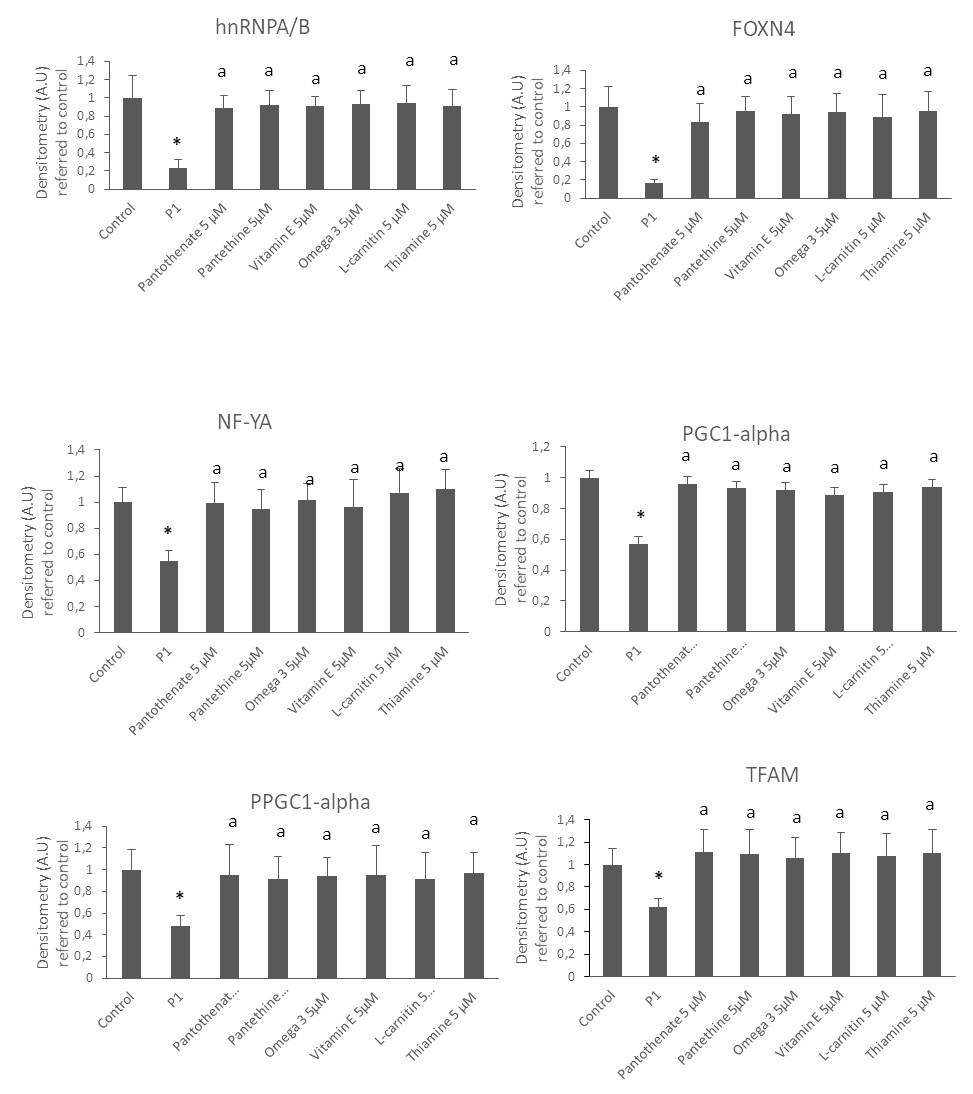


**Supplementary Figure 5.** Densitometry Westerns Figure **6b**. Data represent the mean±SD of three separate experiments. *p<0.01 between PKAN patients and controls. ^a^p<0.01 between untreated and treated fibroblasts. A.U., arbitrary units.

**
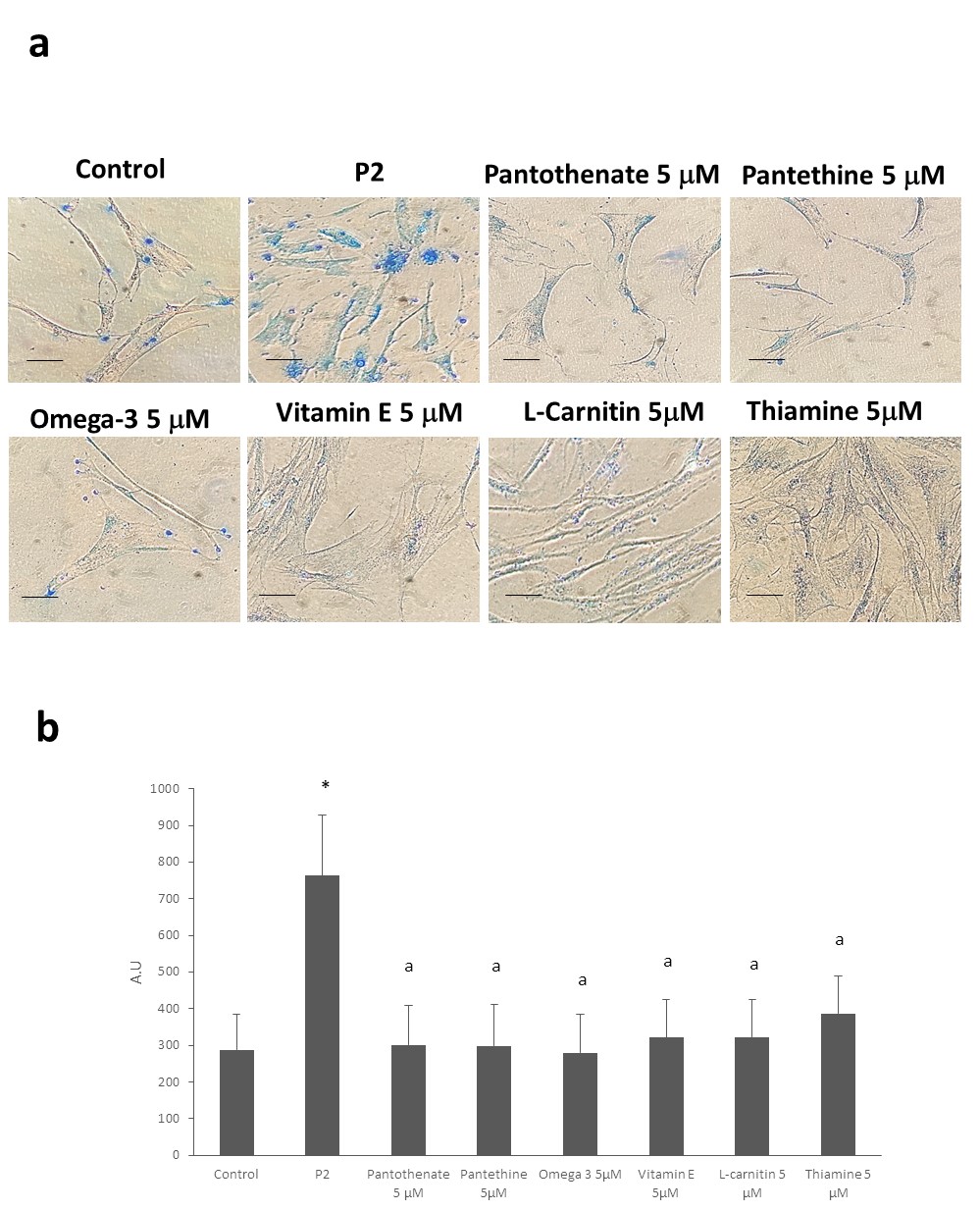
**

**Supplementary Figure 6.** (**a**) Control (C1) and PKAN fibroblasts (P2) were treated with pantothenate, pantethine, vitamin E, omega 3, L-carnitine or thiamine at 5 μM for 20 days. Then, cells were stained with Prussian Blue as described in material and Methods and examined by bright-field microscopy. Scale bar= 15 μm. (**b**) Quantification of Prussian Blue staining. Images were analyzed by the Image J software. *p<0.01 between Control and PKAN fibroblasts. ^a^p<0.01 between untreated and treated fibroblasts. A.U., arbitrary units.

**
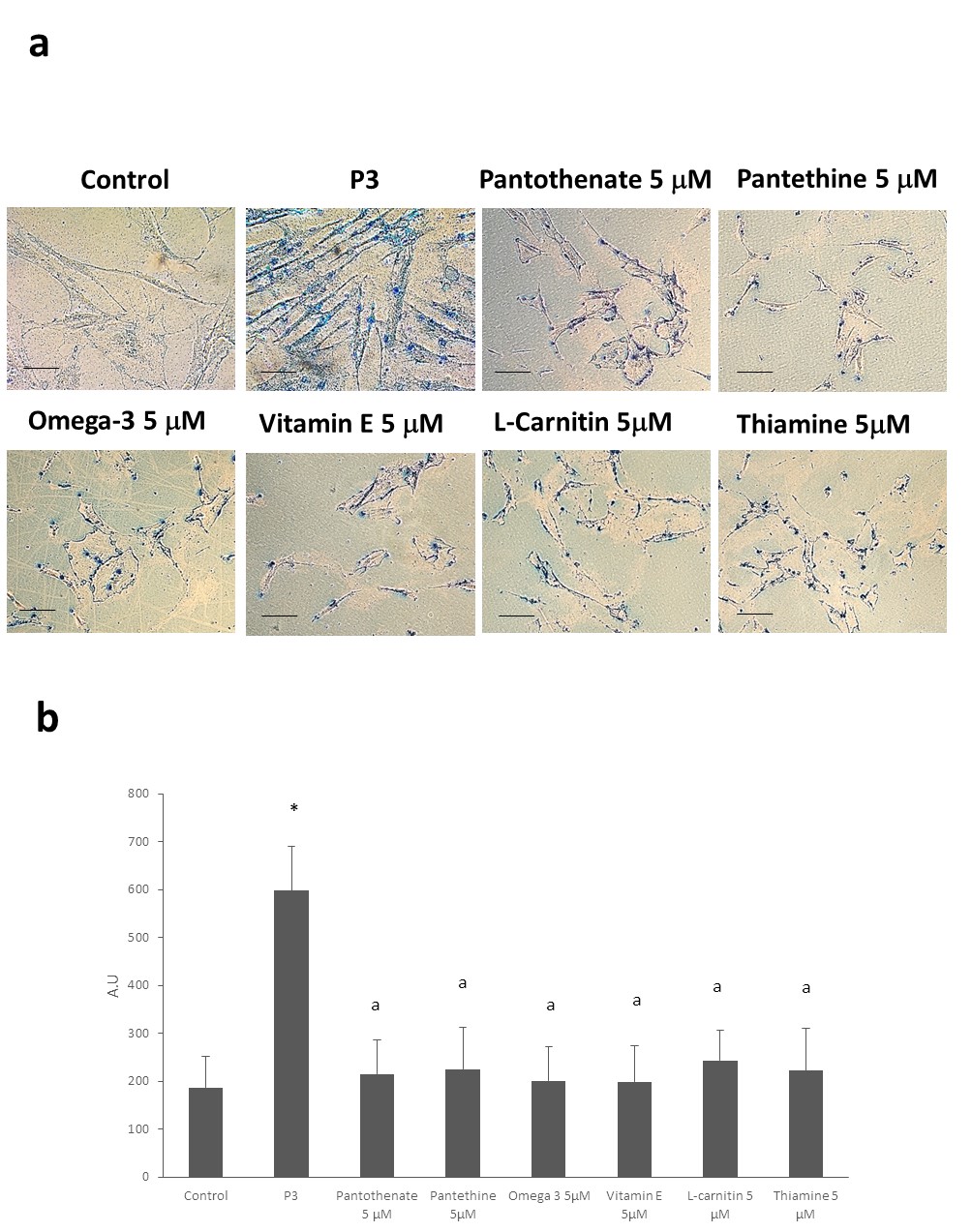
**

**Supplementary Figure 7.** (**a**) Control (C1) and PKAN fibroblasts (P3) were treated with pantothenate, pantethine, vitamin E, omega 3, L-carnitine or thiamine at 5 μM for 20 days. Then, cells were stained with Prussian Blue as described in material and Methods and examined by bright-field microscopy. Scale bar= 15 μm. (**b**) Quantification of Prussian Blue staining. Images were analyzed by the Image J software. *p<0.01 between Control and PKAN fibroblasts. ^a^p<0.01 between untreated and treated fibroblasts. A.U., arbitrary units.

**
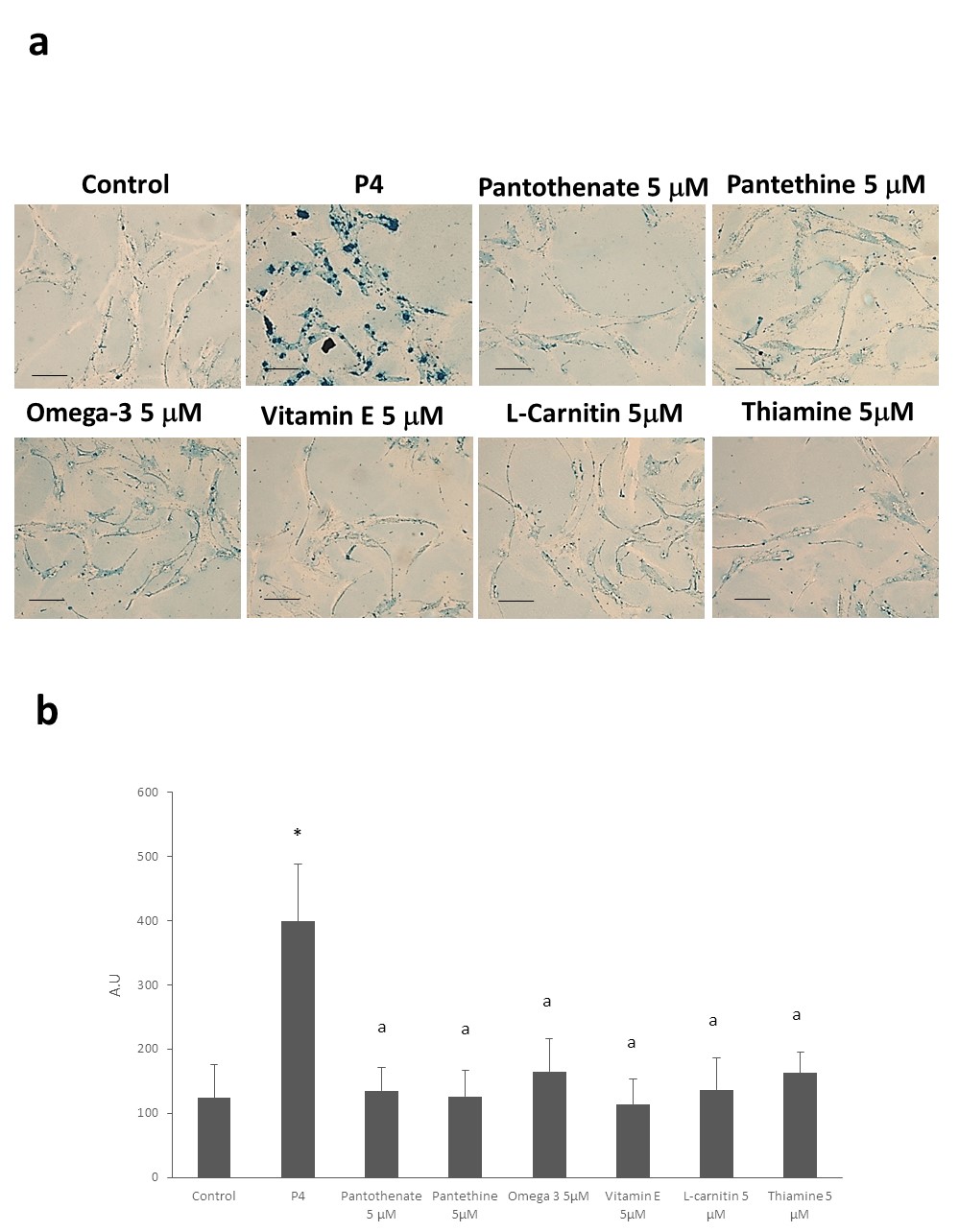
**

**Supplementary Figure 8.** (**a**) Control (C1) and PKAN fibroblasts (P4) were treated with pantothenate, pantethine, vitamin E, omega 3, L-carnitine or thiamine at 5 μM for 20 days. Then, cells were stained with Prussian Blue as described in material and Methods and examined by bright-field microscopy. Scale bar= 15 μm. (**b**) Quantification of Prussian Blue staining. Images were analyzed by the Image J software. *p<0.01 between Control and PKAN fibroblasts. ^a^p<0.01 between untreated and treated fibroblasts. A.U., arbitrary units.


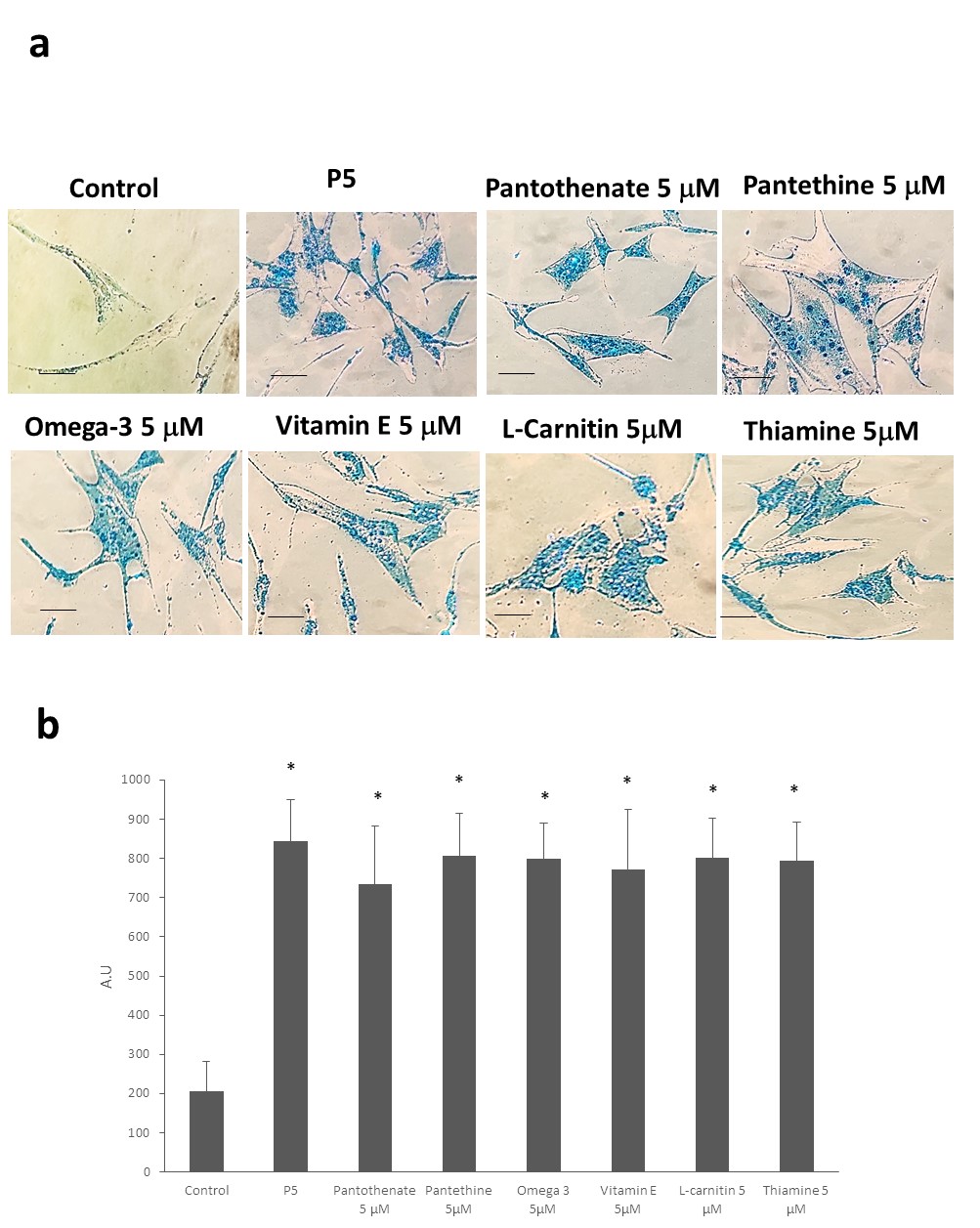


**Supplementary Figure 9.** (**a**) Control (C1) and PKAN fibroblasts (P5) were treated with pantothenate, pantethine, vitamin E, omega 3, L-carnitine or thiamine at 5 μM for 20 days. Then, cells were stained with Prussian Blue as described in material and Methods and examined by bright-field microscopy. Scale bar= 15 μm. (**b**) Quantification of Prussian Blue staining. Images were analyzed by the Image J software. *p<0.01 between Control and PKAN fibroblasts. ^a^p<0.01 between untreated and treated fibroblasts. A.U., arbitrary units.
